# Ferroptosis as a therapeutic target for inflammation-related intestinal diseases

**DOI:** 10.3389/fphar.2023.1095366

**Published:** 2023-01-13

**Authors:** Xiaoli Zhang, Yiming Ma, Guoqing Lv, Hongying Wang

**Affiliations:** ^1^ State Key Laboratory of Molecular Oncology, National Cancer Center/National Clinical Research Center for Cancer/Cancer Hospital, Chinese Academy of Medical Sciences and Peking Union Medical College, Beijing, China; ^2^ State Key Laboratory of Explosion Science and Technology, Beijing Institute of Technology, Beijing, China; ^3^ Department of Gastrointestinal Surgery, Peking University Shenzhen Hospital, Shenzhen Peking University-The Hong Kong University of Science and Technology Medical Center, Guangdong, China

**Keywords:** cell death, ferroptosis, inflammation, inflammatory bowel disease, colorectal cancer

## Abstract

Ferroptosis is an iron-dependent programmed cell death characterized by reactive oxygen species-induced lipid peroxidation and resultant membrane damage. Recent research has elucidated the mechanism of ferroptosis and investigated the relationship between ferroptosis and various diseases, including degenerative diseases, cancer, and inflammation. Ferroptosis is associated with inflammation-related intestinal diseases such as colitis and colitis-associated cancer. New insights into the role of ferroptosis in the pathogenesis of inflammation-related gut diseases have suggested novel therapeutic targets. In this review, we summarize current information on the molecular mechanisms of ferroptosis and describe its emerging role and therapeutic potential in inflammation-related intestinal diseases.

## 1 Introduction

Programmed cell death (PCD) is an evolutionarily conserved process that plays an important role in various physiological processes, including embryonic development and tissue homeostasis (Yang and Stockwell, 2016). Dysregulated PCD machinery is generally accompanied by dysfunction of cells and tissues and is the hallmark of multiple pathological processes, including cancer, inflammation and related diseases (Van Opdenbosch and Lamkanfi, 2019). Over the past decades, several PCD processes regulated through various molecular mechanisms have been identified. The most well-studied form of PCD is apoptosis, which is triggered mainly by the activation of caspase proteases (Günther et al., 2013). Recently, non-apoptotic cell death, such as necroptosis and pyroptosis, has attracted widespread attention (Yu et al., 2021). Ferroptosis, first recognized in 2003, is characterized by the peroxidation of polyunsaturated fatty acids (PUFAs) *via* iron- and reactive oxygen species (ROS)-dependent mechanisms (Dixon et al., 2012; Yang and Stockwell, 2016; Stockwell, 2022). Accumulative lipid peroxidation increases membrane permeability, leading to cell death. Ferroptosis does not involve DNA fragmentation or caspase activation, and can be inhibited by iron chelating agents or antioxidants rather than caspase inhibitors (Dixon et al., 2012). Ferroptosis differs from other types of PCD in terms of morphology, biochemistry, and genetics. Although the physiological function of ferroptosis is poorly understood, its involvement in multiple human diseases, such as degenerative diseases, ischemia-reperfusion injury, tumorigenesis, and inflammatory diseases, has been widely reported (Tang et al., 2021).

Inflammation is a host defense mechanism to kill pathogens, and remove or heal tissue damage (Medzhitov, 2008). In response to tissue injury, cytokine and chemokine networks initiate and maintain an inflammatory process that involves the activation and recruitment of neutrophils, monocytes, eosinophils and mast cells to the damage site. Inflammation is also a consequence of initial neoplastic changes. Activated inflammatory cells release reactive oxygen species (ROS), reactive nitrogen intermediates (RNIs), cytokines, proteases, and other inflammatory mediators resulting in oxidative stress, oxidative damage, and lipid peroxidation (LPO). Dysregulation of inflammatory response or persistent inflammatory insults leads to chronic inflammation, which increases the incidence of cancer. Inflammation has been implicated in the initiation and progression of gastrointestinal (GI) cancers. Inflammatory bowel disease (IBD), including ulcerative colitis (UC) and Crohn’s disease (CD), is a chronic inflammation of the intestinal tract and a high-risk factor for colorectal carcinoma (CRC). Blocking ferroptosis alleviates clinical symptoms of colitis, but promoted colon tumorigenesis, indicating a dual role of ferroptosis in intestinal diseases, such as IBD and CRC (Chen Y et al., 2020; Xu et al., 2020). As ferroptosis appears to play a key role in the pathophysiology of inflammatory gut diseases, it may be a potential therapeutic target.

This review presents a comprehensive overview of ferroptosis, including its features and molecular mechanisms, its role in cellular inflammation and inflammatory intestinal diseases, and its relevance in the development of novel therapeutic targets.

## 2 An overview of ferroptosis

### 2.1 The characteristics of ferroptosis

Ferroptosis is morphologically, biochemically, and genetically distinct from other forms of programmed cell death, such as apoptosis, necroptosis, pyroptosis, and autophagy-dependent cell death, and is characterized by morphological changes, iron overload, ROS accumulation, and lipid peroxidation (Vanden Berghe et al., 2014).

#### 2.1.1 Morphological changes

Cells undergoing ferroptosis show a necrotic morphology, including lack of chromatin condensation, increased membrane density, and ruptured outer cell membrane (Hassannia et al., 2019). Plasma-membrane damage leading to ferroptosis is associated with the formation of membrane nanopores of a few nanometers in radius, followed by bursting of the cell (Pedrera et al., 2021). At the ultrastructural level, transmission electron microscopy has revealed that ferroptotic cells usually exhibit mitochondrial condensation or swelling, increased bilayer membrane density, and reduced or absent mitochondrial crista (Tang et al., 2021). Mitochondrial abnormalities indicate that mitochondria-associated ROS production and metabolic changes are required for ferroptosis.

#### 2.1.2 Iron overload

As is evident from the name, ferroptotic cell death is closely related to iron. Iron levels, particularly that of ferrous iron, are significantly elevated in various pathological processes involving ferroptosis, such as hemorrhagic brain and ulcerative colitis (Li et al., 2017; Xu et al., 2020). Our previous study shows that ferroptosis is involved in intestinal epithelial cell death during colitis-associated carcinogenesis and is accompanied by elevated ferrous iron level in colon tissues (Zhang et al., 2021). Moreover, the classical ferroptosis inducers erastin or 1S, 3R-RSL3 (RSL3) can increase intracellular iron (Dixon et al., 2012). Iron is a redox-active metal that can directly generate excessive ROS through the Fenton reaction and thereby promote lipid peroxidation. Iron may also increase the activity of lipoxygenases (LOXs), which are non-heme iron-containing enzymes responsible for lipid peroxidation (Jiang et al., 2021). Therefore, elevated iron levels can increase the vulnerability to ferroptosis, and loading iron into tumor cells *via* nanoparticles induces ferroptosis, whereas iron chelators prevent ferroptosis. Various genes or proteins involved in iron homeostasis including import, export, storage, and turnover, affect ferroptosis sensitivity (Doll and Conrad, 2017). An unresolved question is why other metal ions generating ROS fail to induce ferroptosis, and is worth further study in the future.

#### 2.1.3 Lipid peroxidation

**FIGURE 1:**
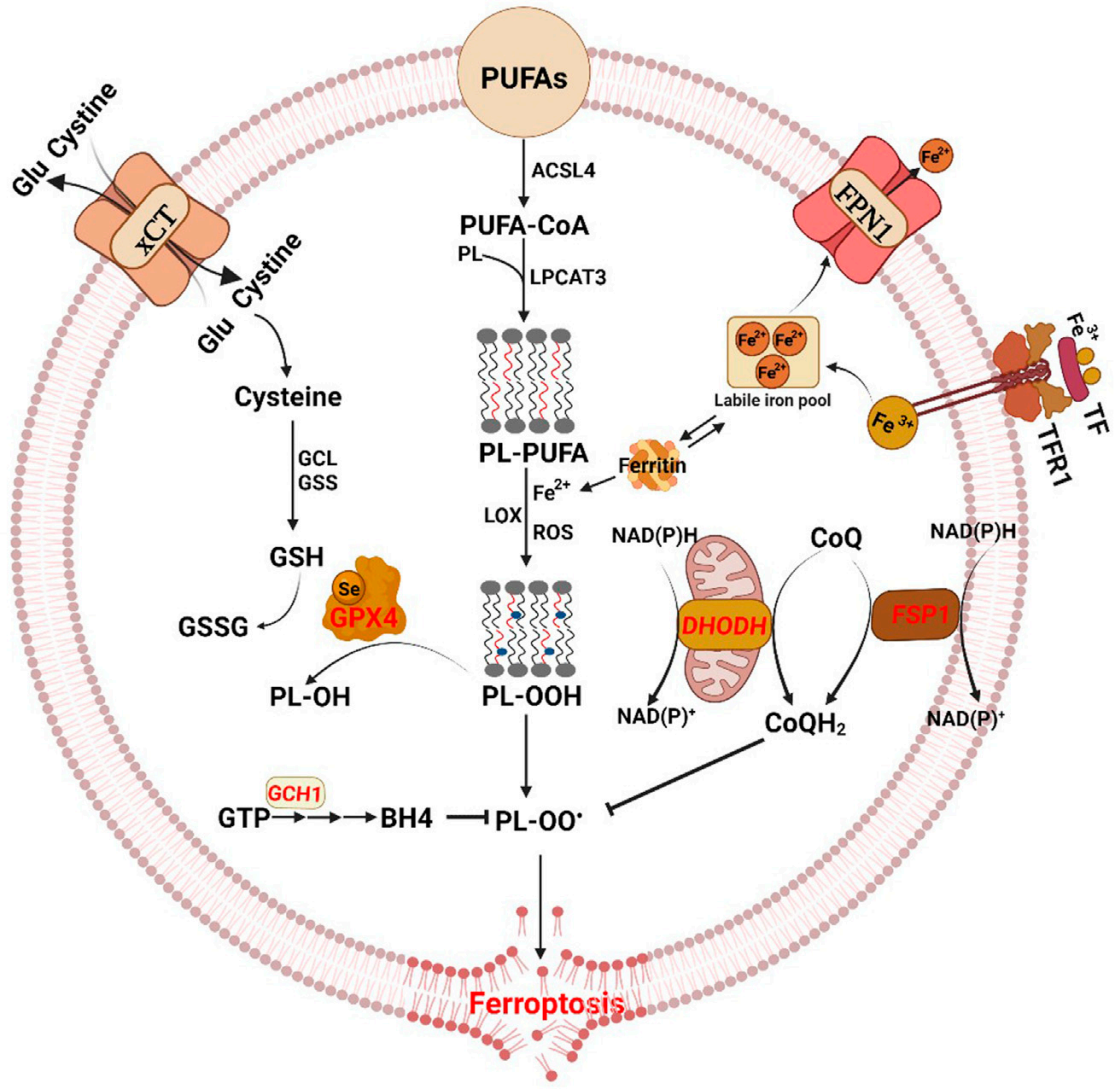
The antioxidant pathways of ferroptosis. The diagram shows the core process of ferroptosis and key molecules. Ferroptosis is characterized by iron load, excessive reactive oxygen species (ROS) production and lipid peroxidation accumulation. The three main regulatory mechanisms of ferroptosis, with different subcellular localization, include the cytoplasmic cyst(e)ine/GSH/GPX4 pathway, the cell membrane-associated FSP1–CoQ-NAD(P)H pathway, and the mitochondrial DHODH–CoQ-NAD(P)H pathway. Moreover, there are additional regulators of ferroptosis. Glu glutamate, GCL glutamate-cysteine ligase, GSS glutathione synthetase, GSH glutathione, GSSG oxidized glutathione, GPX4 glutathione peroxidase 4, PUFAs polyunsaturated fatty acids, ACSL4 acyl-CoA synthetase long-chain family member 4, PL phospholipid, LPCAT3 lysophosphatidylcholine acyltransferase 3, LOX lipoxygenase, FSP1 ferroptosis suppressor protein 1, DHODH dihydroorotate dehydrogenase, TF transferrin, TFR1 transferrin receptor protein-1, FPN1 ferroportin 1, GTP guanosine triphosphate, GCH1 GTP cyclohydrolase 1, BH4 tetrahydrobiopterin.

The essential role of esterified PUFAs in ferroptosis was corroborated by the replacement of natural PUFAs with deuterated PUFAs (Yang et al., 2016). PUFAs, which contain bis-allylic hydrogen atoms that can be readily abstracted, are susceptible to lipid peroxidation (Yang et al., 2016). In general, arachidonic acid (AA)- and adrenic acid (AdA)- containing phosphatidylethanolamines (PE) were vulnerable to ROS attack and are, as such, the prime substrates for lipid peroxidation. These long-chain PUFAs are preferentially catalyzed into their acyl-CoA esters by acyl-CoA synthetase long-chain family member 4 (ACSL4), and re-acylated into lysophospholipids by lysophosphatidylcholine acyltransferase 3 (LPCAT3), and subsequently oxidized by lipoxygenase, which results in membrane rupture and ferroptotic cell death (Figure 1) (Doll et al., 2017). The products of lipid peroxidation, including the initial lipid hydroperoxides and subsequent reactive aldehydes and 4-hydroxynonenal, are increased during ferroptosis, ultimately destabilize the cell membrane, leading to pore formation and ferroptosis (Pedrera et al., 2021). Supplementing cells with PUFAs promotes ferroptosis, whereas monounsaturated fatty acids (MUFAs) suppress ferroptosis by inhibiting lipid peroxidation (Tesfay et al., 2019). In line with this, we find that the MUFA oleic acid suppressed ferroptosis (Zhang et al., 2021), partly by displacing PUFAs from plasma membrane phospholipids. In addition, a high fat diet (HFD), which induces systemic accumulation of lipids and their metabolites, could repress ferroptosis in the intestinal epithelial cells (IECs) (Zhang et al., 2022a), indicating the necessity of distinguishing the effect of different lipid components of the diet on ferroptosis.

#### 2.1.4 Genetics features

A serious of genetic markers, such as aldo-keto reductase family 1 member C1 (AKR1C1), ChaC glutathione specific gamma-glutamylcyclotransferase 1 (CHAC1), ferritin heavy chain 1 (FTH1), prostaglandin endoperoxide synthase 2 (PTGS2), are associated with cells undergoing ferroptosis (Stockwell et al., 2017). These changes may not be observed at the same time in all experiments. AKR1C1, an aldo-keto reductase enzyme involved in steroid metabolism, can prevent ferroptosis by reducing lipid peroxidation end-products to their corresponding non-toxic lipid-derived alcohols, and is dramatically upregulated in ferroptotic cells concomitantly (Gagliardi et al., 2019). The expression of CHAC1, which digests glutathione (GSH) into 5-oxoproline and cysteine-glycine dipeptide, was induced by cystine starvation-triggered ferroptosis (Chen et al., 2017). FTH1, a functional subunit of the iron-storage protein ferritin, maintains intracellular free iron levels to limit iron toxicity, and are generally upregulated during ferroptosis (Xu et al., 2020). PTGS2, the key enzyme in prostaglandin biosynthesis, is induced during ferroptosis as well (Stockwell, 2022). Due to the inability of PTGS2 to directly oxidize lipids, it is usually considered as a biomarker, but not as a driver, of ferroptosis (Tang et al., 2021). In addition, RSL3 can elevate the AKR1C1, CHAC1, FTH1, and PTGS2 expression in various cell lines (Zhang et al., 2021).

### 2.2 Antioxidant system in ferroptosis

As ferroptosis is a result of metabolic dysfunction involving ROS, iron, and PUFAs, various genes and pathways related to iron metabolism, lipid synthesis, and antioxidant systems can potentially mediate ferroptosis (Hassannia et al., 2019). This review focuses on the involvement of the anti-lipid oxidation system, which is closely associated with inflammation.

#### 2.2.1 Cytoplasmic cyst(e)ine/GSH/GPX4 and GCH1-BH4 pathways

Ferroptosis is characterized by depletion of GSH and decreased expression of GPX4. The cyst(e)ine/GSH/GPX4 axis is considered the main system suppressing ferroptosis (Figure 1) (Ursini and Maiorino, 2020). GSH is a thiol-containing tripeptide that plays an essential role in intracellular antioxidant defense and ferroptosis prevention. The biosynthesis of GSH requires the participation of glutamate, glycine, and cysteine which is the rate-limiting substrate and produced from cystine imported by the cystine/glutamate antiporter system X_c_^−^ (xCT) (Hassannia et al., 2019). GPX4, a member of the glutathione peroxidase family, converts glutathione into oxidized glutathione (GSSG) and reduces the cytotoxic lipid peroxides (L-OOH) to the corresponding alcohols (L-OH) (Yang et al., 2014). Therefore, system X_c_^−^ inhibition, GSH depletion or GPX4 inactivation cause increased oxidative stress and subsequent ferroptosis. Erastin and sulphasalazine are inhibitors of system X_c_^−^ and decrease GSH levels, thereby inducing ferroptosis. RSL3 and FIN56 are direct inhibitors of GPX4 active sites, thereby triggering ferroptosis (Stockwell et al., 2017). Although GPX4 overexpression in cells confers resistance to ferroptosis, and GPX4 knockdown promotes ferroptosis, a prevailing role for GPX4 in ferroptosis has been questioned. Cancer drug-tolerant persister cells are vulnerable to GPX4 inhibition because of the upregulated GPX4, which has been identified as a likely target molecule (Wang et al., 2021b; Zhang et al., 2022b).

Although cyst(e)ine/GSH/GPX4 axis is the best-researched pathway in ferroptosis regulation, GPX4-independent mechanisms of ferroptosis surveillance have also been identified. The GTP cyclohydrolase-1 (GCH1)-tetrahydrobiopterin (BH4) metabolism acts as an endogenous antioxidant pathway to inhibit ferroptosis through a mechanism independent of the GSH/GPX4 system (Wei et al., 2020). BH4, a redox-active cofactor, exhibits antioxidant properties and is synthesized by GCH1 catalyzing GTP (Soula et al., 2020). Overexpression of GCH1 or supplementation of ferroptotic cells with BH4 yields almost complete protection against ferroptosis through abolishing lipid peroxidation (Kraft et al., 2020). Moreover, the GCH1-BH4 pathway selectively protects cells against ferroptosis but not apoptosis (Kraft et al., 2020).

#### 2.2.2 Membrane-associated FSP1–CoQ-NAD(P)H pathway

Ferroptosis suppressor protein 1 (FSP1), previously known as apoptosis-inducing factor mitochondria-associated 2 (AIFM2), was shown to block lipid peroxidation and suppress ferroptosis by regenerating reduced coenzyme-Q10 (CoQ10) in a GPX4-or GSH-independent manner (Bersuker et al., 2019; Doll et al., 2019). The N-terminus of FSP1 contains a canonical myristoylation motif associated with lipid bilayers, and mutation of the myristoylation site prevents its antiferroptotic function. Myristoylated FSP1 is recruited to the plasma membrane and produces ubiquinol, a reduced form of CoQ10, using NAD(P)H, which protects against lipid peroxidation (Figure 1). CoQ10 suppresses lipid peroxidation by capturing radical intermediate functions outside of the mitochondria. Thus, depletion of CoQ10 sensitizes cells to ferroptosis. FSP1-CoQ10-NAD(P)H pathway may serve as a parallel system to the GSH/GPX4 axis. However, some studies have also found that FSP1 can block ferroptosis through an ubiquinol-independent mechanism (Dai et al., 2020b). FSP1-dependent endosomal sorting complexes required for transport (ESCRT)-III recruitment in the plasma membrane is responsible for ferroptosis inhibition through the activation of a membrane repair mechanism (Dai et al., 2020b). There results indicate that FSP1 can inhibit ferroptosis through multiple mechanisms, and the need for further studies on the unknown mechanisms of FSP1 in the regulation of ferroptosis.

#### 2.2.3 Mitochondrial DHODH–CoQ-NAD(P)H pathway

In addition to the suppression of ferroptosis *via* GPX4, which utilizes reduced GSH to detoxify lipid hydroperoxides and inhibits ferroptosis in cytoplasm, and FSP1, which functions as an oxidoreductase to reduce CoQ to CoQH2 mainly on the plasma membrane, the mitochondrial DHODH–CoQ-NAD(P)H pathway is an additional cellular defense mechanism against ferroptosis (Figure 1) (Mao et al., 2021)**.** Dihydroorotate dehydrogenase (DHODH) catalyzes the conversion of dihydroorotate to orotate and reduces ubiquinone (CoQ) to ubiquinol within the mitochondria, therby preventing ferroptotic cell death (Stockwell, 2022). DHODH inactivation induces extensive mitochondrial lipid peroxidation and ferroptosis in GPX4^low^ cells. The mitochondrial DHODH-mediated ferroptosis defense mechanism may be exploited as a therapeutic target.

## 3 The interaction between inflammation and ferroptosis

### 3.1 Inflammation promotes ferroptosis

**FIGURE 2:**
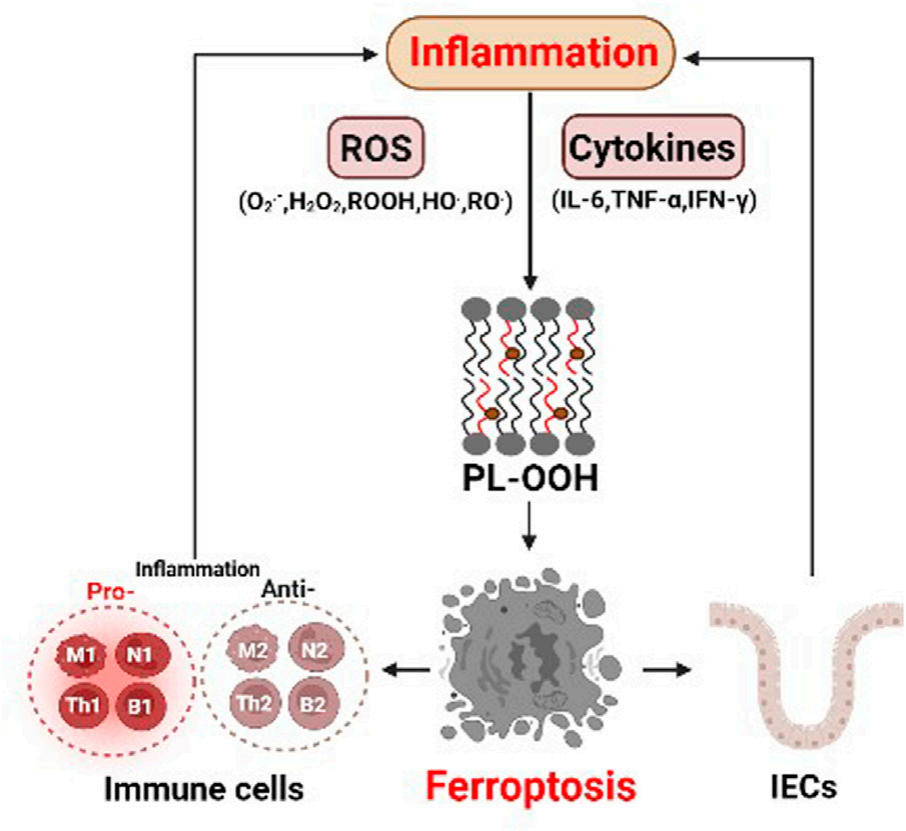
The interaction between ferroptosis and inflammation. Abundant reactive oxygen species (ROS) in inflammation can lead to lipid peroxidation and ferroptosis. In addition, various cytokines, such as IL-6, TNF-α and IFN-γ, may play critical roles in inducing ferroptosis. The pro-inflammatory and anti-inflammatory cells themselves both can undergo ferroptosis, which directly affects inflammatory responses. intestinal epithelial cells (IECs) intestinal epithelial cells.

ROS are extremely abundant in the inflammatory process, and mainly include superoxide (O_2_^.-^), peroxides (H_2_O_2_ and ROOH) and free radicals (HO^.^ and RO^.^) (Spooner and Yilmaz, 2011). An imbalance between ROS and antioxidants can cause oxidative stress. On the one hand, the oxidative stress can promote the expression of pro-inflammatory genes, including interleukin-6 (IL-6) and tumor necrosis factor (TNF-α), by activating key transcription factors, such as NF-κB (Hussain et al., 2016; Neganova et al., 2021). On the other hand, the oxidative stress can damage DNA, proteins and lipids, leading to excessive lipid peroxidation, a key event in ferroptosis (Figure 2).

In addition, immune cells release inflammatory mediators, such as TNF-α, IL-6 and interferon gamma (IFN-γ), which might influence ferroptosis (Figure 2). Typically, IL-6, a marker of M1 macrophages, promotes lipid peroxidation in bronchial epithelial cells by increasing ferrous ion levels, thereby inducing ferroptosis (Han et al., 2021). Stimulation of TNF-α, a well-known inducer of receptor-dependent apoptosis under inflammatory condition (Brenner et al., 2015), increases the expression of acyl-CoA synthetase long-chain family member 1 (ACSL1) in human endothelial cells, which promotes ferroptosis by enhancing the synthesis of polyunsaturated lipids and subsequent lipid peroxidation (Jung et al., 2020). IFN-γ released from CD8^+^ T cells can downregulate the expression of cysteine transporters SLC3A2 and SLC7A11, consequently promoting lipid peroxidation and ferroptosis in tumor cells (Wang et al., 2019). These results indicate that inflammatory cytokines may play a critical role in inducing ferroptosis.

### 3.2 Ferroptosis affects inflammation through immunogenicity

**FIGURE 3:**
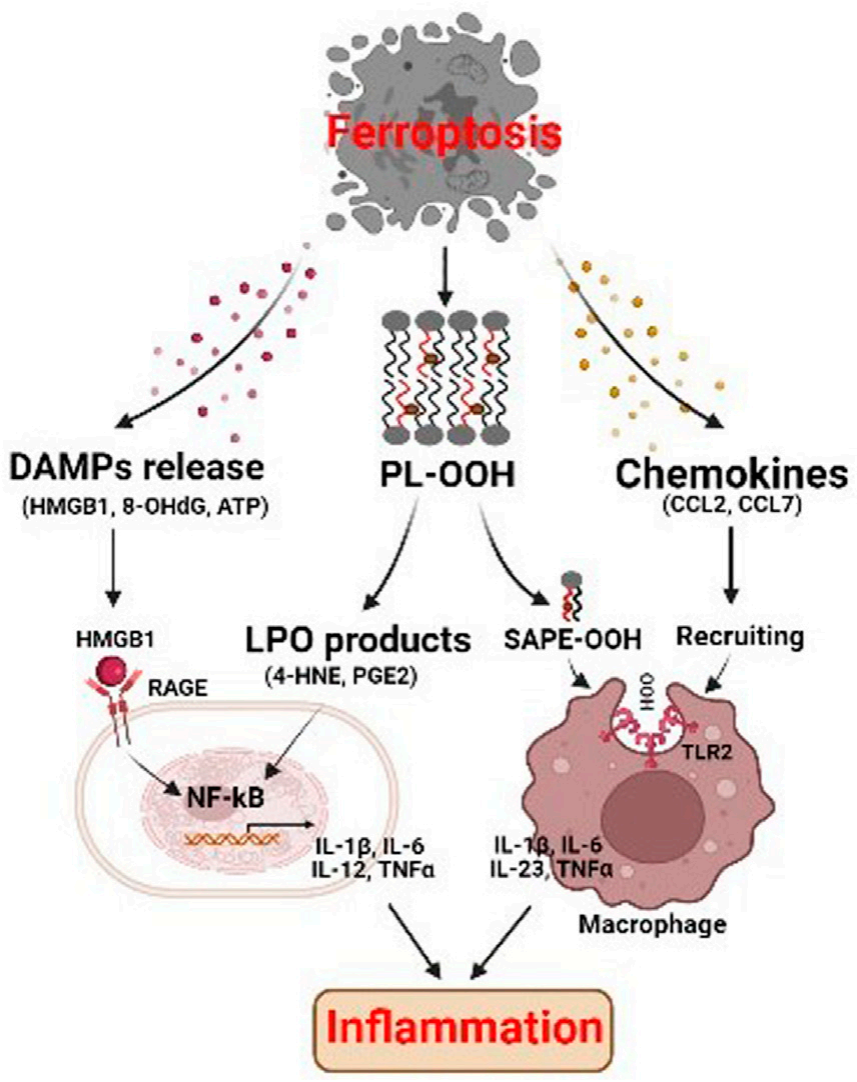
Ferroptosis affects inflammation through immunogenicity. The ferroptotic cells release damage-associated molecular patterns (DAMPs) and lipid peroxidation products, which may promote inflammatory response through activating NF-kB pathway. SAPE-OOH on ferroptotic cell surface, the oxidized phospholipid, acts as an eat-me signal and navigates phagocytosis by targeting TLR2 on macrophages. Ferroptotic cells can also secrete chemokines which induce the recruitment of macrophages, and cause inflammation. HMGB1 high-mobility group protein B1, RAGE receptor for advanced glycation end-products, NF-kB nuclear factor kappa B, PL-OOH phospholipid-OOH, 4-HNE 4-hydroxynonenal, PGE2 prostaglandin E2, SAPE-OOH 1-steaoryl-2-15-HpETE-sn-glycero-3phosphatidylethanolamine, TLR2 toll like receptor 2.

Cell death is not only a consequence of inflammation but can also promote an inflammatory response. Upon plasma membranes ruptured, ferroptotic cells release intracellular components that function as danger signals for the innate immune system. These signals include lipid peroxidation (LPO) products, such as oxidized phospholipids (oxPLs), 4-hydroxynonenal (4-HNE) and prostaglandin E2 (PGE2), and damage-associated molecular patterns (DAMPs), such as high-mobility group protein B1 (HMGB1), DNA, and ATP. For example, 1-steaoryl-2-15-HpETE-sn-glycero-3phosphatidylethanolamine (SAPE-OOH), an oxidized PL on the surface of ferroptotic cells, serves as an eat-me signal, which recruits macrophages to clear the dying cells (Figure 3) (Luo et al., 2021). The lipid peroxidation product 4-HNE is a pro-inflammatory mediator that activates the NF-κB pathway in chronic disease (Jang et al., 2016). Ferroptosis is also associated with increased PTGS2 expression and PGE2 release, which is the key inflammatory cytokine that plays important and complex roles in inflammation (Yang et al., 2014). As for DAMPs, they bind to specific receptors to activate inflammation and initiate highly optimized sequences of immune cell recruitment to trigger effective tissue repair (Gong et al., 2020). Ferroptotic cells can release HMGB1, which promotes inflammation and development of numerous inflammatory diseases through NF-κB activation, mediated by its receptor advanced glycosylation end-product specific receptor (AGER) (Pandolfi et al., 2016). In addition, released HMGB1 can promote M1 polarization of macrophages *via* the HMGB1-AGER signaling pathway (Wen et al., 2019). HMGB1 inhibition can therefore be used to treat ferroptosis-related inflammation. Moreover, ferroptotic cells can release 8-hydroxy-2′-deoxyguanosine (8-OHdG), a major product of oxidative DNA damage. Released 8-OHdG activates the stimulator of interferon genes (STING)-dependent DNA sensor pathway in macrophages, resulting in its further infiltration (Dai et al., 2020a). Ferroptotic cells can also secrete chemokines, such as CCL2, CCL7, which induce the recruitment of macrophages, and cause inflammation (Wang et al., 2021a).

### 3.3 Ferroptosis of inflammatory cells

Immune cells are recruited to the inflammatory site, where they secret various cytokines and chemokines or clear the bacteria and dead host cells through phagocytosis. Immune cells themselves can undergo ferroptosis (Figure 2) (Chen et al., 2021). Macrophages, for example, can polarize their functions on a continuum between pro-inflammatory M1 macrophages, and anti-inflammatory M2 macrophages (Murray, 2017). Although the expression levels of ACSL4, LPCAT3, and GPX4 were similar in M1 and M2 macrophages, the M1 subtype exhibited higher resistance to ferroptosis and promoted inflammatory responses (Kapralov et al., 2020). Mechanistically, M1 cells exhibit the loss of arachidonate 15 lipoxygenase activity, and reduced lipid peroxidation (Xu H et al., 2021). In addition to macrophages, neutrophil granulocytes are also the first immune cells recruited to inflammation sites, where they fight pathogens through phagocytosis, degranulation, and the release of neutrophil extracellular traps (NETs) coupled to the death of the cells. Recent studies have shown that ferroptosis is associated with the formation of NETs (Zou et al., 2020). Moreover, neutrophil deaths in systemic lupus erythematosus patients, a large part show ferroptosis due to the reduced GPX4 (Ohl et al., 2021). B cells, including B1 and B2, are responsible for the synthesis of antibodies and inflammatory response adaptation through secretion of IL-2, IFN-γ and TGF-β (Kałużna et al., 2022). Ferroptosis is involved in the development and homeostasis of innate-like B1-cells, which regulate efficient antibody responses to bacteria (Chen et al., 2022). As B1 cells express higher CD36 levels and have higher lipids levels than B2 cells, B1-cells differentiation is more dependent on GPX4 and more sensitive to lipid peroxidation and ferroptosis induced by GPX4 deletion (Muri et al., 2019).

## 4 Ferroptosis and inflammatory bowel disease

**FIGURE 4:**
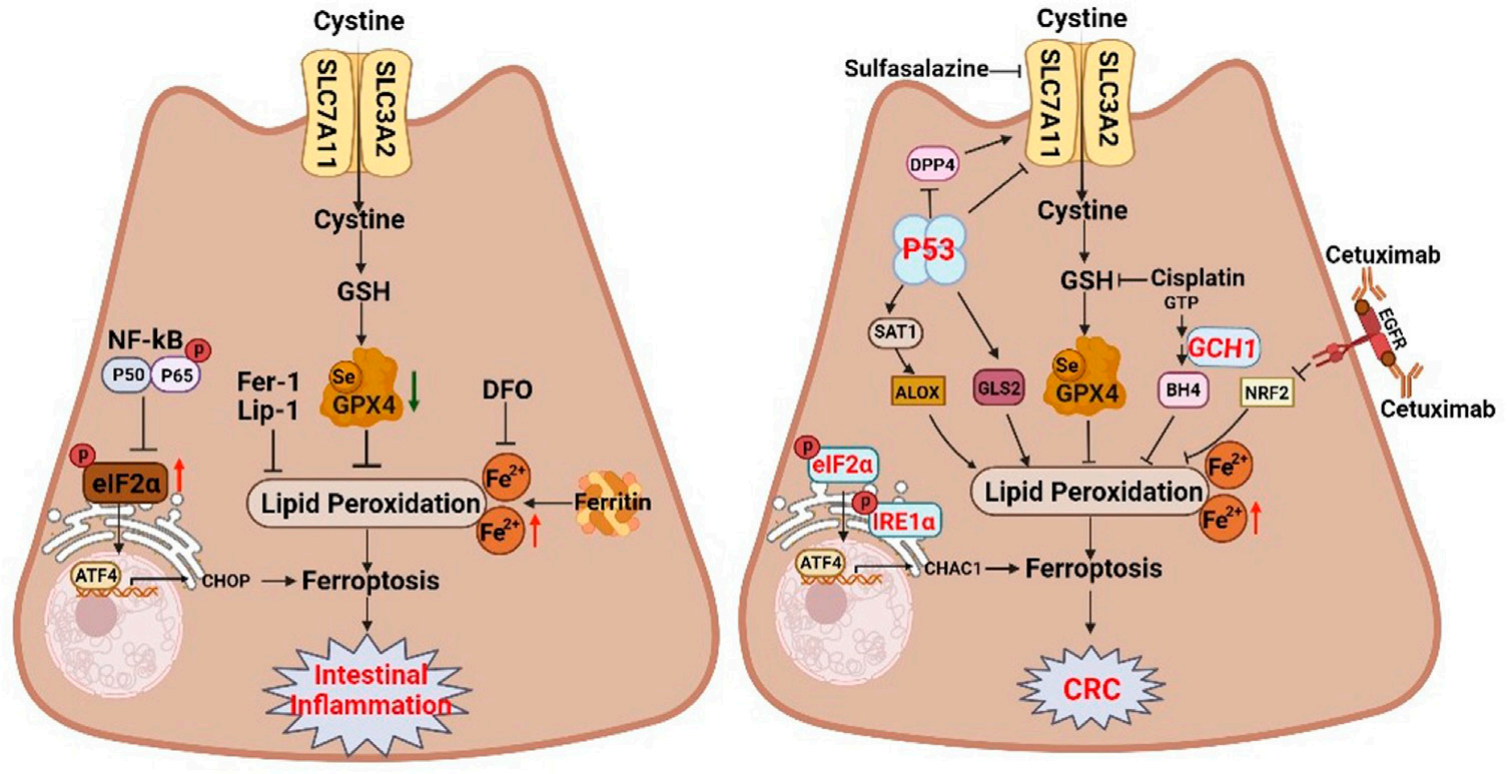
The dual regulatory roles of ferroptosis in inflammatory bowel disease and colorectal cancer. Ferroptosis inhibition can attenuate intestinal injury in intestinal inflammation. However, inducing ferroptosis with pharmacological activators can inhibit the proliferation of colorectal neoplasms. The schematic diagram shows related ferroptosis regulators and pathways. SLC7A11 solute carrier family 7 member 11, SLC3A2 solute carrier family 3 member 2, GSH glutathione, GPX4 glutathione peroxidase 4, NF-kB nuclear factor kappa B, eIF2α eukaryotic translation initiation factor 2 subunit alpha, ATF4 activating transcription factor 4, CHOP DNA damage inducible transcript 3, DFO deferoxamine, Fer-1 ferrostatin-1, Lip-1 Liproxstatin-1, IRE1α inositol-requiring enzyme 1α, CHAC1 ChaC glutathione specific gamma-glutamylcyclotransferase 1, DPP4 dipeptidyl peptidase 4, SAT1 spermidine/spermine N1-acetyltransferase 1, ALOX arachidonate lipoxygenase, EGFR epidermal growth factor receptor, NRF2 NFE2 like bZIP transcription factor 2.

IBD is a chronic inflammation of the intestinal tract, characterized by a reduction in crypts, villus atrophy, and inflammation of the intestinal mucosa (Li et al., 2021). Abnormal cell death of IECs compromises intestinal barrier and aggravates the inflammatory response. In addition to apoptosis, necroptosis, and pyroptosis, ferroptotic cell death has been observed in IECs at the inflamed sites in patients with UC and CD (Xu et al., 2020; Xu C et al., 2021). Elevated levels of ROS and LPO products in colitis provide suggestive evidence for the presence of ferroptosis in these diseases (Wang et al., 2020). IECs isolated from UC patients and mice with colitis exhibited increased PTGS2 and decreased GPX4, which are biomarkers of ferroptosis (Xu et al., 2020). Similarly, we also observe that ferroptosis of IEC is involved in dextran sulphate sodium (DSS)-induced colitis in mice (Zhang et al., 2022a). Importantly, ferrostatin-1 (Fer-1), a specific ferroptosis inhibitor, ameliorates colitis (Chen Y et al., 2020). Consistent with UC, GPX4 activity is also reduced in intestinal epithelial cells from patients with CD (Mayr et al., 2020). Currently, two potential mechanisms have been involved in the ferroptosis of IECs in colitis. First, Xu et al. (2020) showed that ferroptosis contributes to UC *via* endoplasmic reticulum (ER) stress-mediated IEC cell death, which can be alleviated through NF-κBp65 phosphorylation (Figure 4) (Xu et al., 2020). Moreover, some studies indicate that ferroptosis regulates colitis through the Nrf2/HO-1 signaling pathway which can inhibit the NF-κB pathway, and subsequently suppress secretion of the proinflammatory cytokines (Rahman et al., 2021).

Key events of ferroptosis, including iron deposition, lipid peroxidation accumulation, and GPX4 inactivation, are associated with the IBD pathogenesis. For example, iron overload leads to oxidative damage in the gut, resulting in lipid peroxidation accumulation, which probably contributes to IBD pathology (Chieppa et al., 2017). Oral administration of the iron chelator deferiprone promotes intestinal epithelial cell repair and improves clinical symptoms in IBD patients (Figure 4) (Minaiyan et al., 2012). Targeting iron may therefore be a promising therapeutic strategy for IBD. In addition, impaired of GPX4 in IEC of IBD patients implicates GPX4 in gut homoeostasis (Chen Y et al., 2020), and GPX4 activation can markedly inhibit ferroptosis and improve IBD symptoms (Wang et al., 2020). Collaboratively, the pathological role of ferroptosis in IBD suggests ferroptosis as a potential therapeutic target for IBD.

## 5 Ferroptosis and colorectal cancer

Inflammation has been implicated in the initiation and progression of GI cancer. In addition to IBD, CRC progression is closely associated with ferroptosis. CRC is the third-most prevalent cancer and second-most cause of cancer-related deaths in the world (Sung et al., 2021). Colitis-associated cancer (CAC), a subtype of CRC, is associated with intestinal inflammation. Augmented oxidative stress in chronic inflammation causes oxidative damage to macromolecules, contributes to genomic instability, affects cell viability, and promotes carcinogenesis (Sung et al., 2021; Zhang et al., 2021). Our research reveals that ferroptosis is involved in azoxymethane/dextran sulfate sodium (AOM/DSS)-induced tumors in mice, as evidenced by elevated iron levels, lipid peroxidation, and ferroptotic markers, as well as shrunken mitochondria with disrupted cristae (Zhang et al., 2021). Crucially, the ferroptosis-specific inhibitor Fer-1 aggravates colonic tumors (Figure 4) (Zhang et al., 2021). P53, one of the most well-known tumor suppressors, is mutated in approximately 55%–60% of human colorectal cancers, and is mutated in the early stages of CAC (Gao et al., 2021). Interestingly, P53 transcriptionally inhibits the expression of the cystine/glutamate antiporter subunit SLC7A11, thereby suppressing cystine uptake, disrupting GSH biosynthesis, and sensitizing cells to ferroptosis (Figure 4) (Jiang et al., 2015). Therefore, although ferroptosis contributes to mucosa injury in IBD, ferroptosis inhibition promotes CAC.

Tumor growth inhibition by ferroptosis provides new insights into cancer therapies. Elevated iron concentration makes cells more sensitive to ferroptosis. About 60% of CRC patients are iron deficient, and those with iron deficiency have a poorer prognosis and are less responsive to treatment (Wang et al., 2022). Thus, appropriate iron supplementation is of great benefit to the treatment and prognosis of CRC patients (Aksan et al., 2021). The dichloroacetate (DCA) reduces the viability of CRC cells and exhibits anticancer effects by upregulating iron levels (Sun et al., 2021), indicating iron supplementation-induced cellular ferroptosis is beneficial to the treatment of CRC. Moreover, xCT is highly expressed in CRC cell lines and tissues, and can be inhibited by sulfasalazine, ultimately interrupting the synthesis of GSH and resulting in inhibition of the growth of CRC cells (Figure 4) (Ma et al., 2015). The finding suggests that SLC7A11 inhibition may be a potential therapeutic approach for CRC. Currently, some clinical drugs for CRC treatment can inhibited tumor growth by inducing ferroptosis. The classic chemotherapy drug cisplatin can induce ferroptosis, and the combination of cisplatin and erastin synergistically inhibits CRC (Guo et al., 2017; Xu S et al., 2021). In addition, cetuximab, a monoclonal antibody targeting the epidermal growth factor receptor, suppresses NRF2/HO-1 signaling pathway, thereby promoting CRC ferroptotic cell death (Figure 4) (Yang et al., 2021). Moreover, GCH1/BH4 metabolism is also an emerging ferroptosis-resistance mechanism in CRC (Figure 4) (Hu et al., 2022). As blocking GCH1/BH4 promotes erastin-induced ferroptosis, GCH1 inhibitors combined with erastin may be a novel therapeutic strategy for CRC (Hu et al., 2022).

Targeting ferroptosis may, in addition to inhibiting CRC growth, overcome conventional CRC drug resistance. Despite effective chemotherapy and targeted therapy for CRC, drug resistance may result in therapeutic failure and tumor relapse. There is still a lack of precise therapeutic methods for targeting drug-resistant tumor cells. The greater sensitivity of colorectal cancer stem cells (CSCs) to ferroptosis relative to parental CRC cells, can be exploited to combat CRC chemoresistance and progression driven by colorectal CSCs (Izumi et al., 2017). Chen P et al. (2020) demonstrated the anti-tumor effects of combined treatment with the ferroptosis inducer β-elemene and cetuximab, which triggers ferroptosis in CRC patients who do not respond to cetuximab (Chen P et al., 2020). Furthermore, our work reveals that the upregulated GPX4 and ferrous iron seen in anti-colorectal cancer drug-tolerant persister cells may be potential therapeutic targets, and GPX4 inhibition combined with chemotherapy or targeted therapy may be a promising therapy for CRC (Zhang et al., 2022b). Taken together, induction of ferroptosis is a potential strategy for CRC treatment.

## 6 Conclusion and perspectives

We reviewed the morphological, biochemical and genetical characteristics of ferroptosis and the antioxidative regulatory mechanisms of ferroptosis in different subcellular localizations. We also reviewed the relationship between ferroptosis and inflammation, focusing on the role and therapeutic significance of ferroptosis in inflammatory gut diseases, including IBD and CRC. Ferroptosis plays a dual role in various inflammatory intestinal diseases. Inhibiting of ferroptosis can attenuate intestinal injury in IBD; however, induction of ferroptosis inhibits the migration and proliferation of CRC cells. In addition to inflammatory gut diseases, ferroptosis also plays an important role in other inflammation-related diseases, such as non-alcoholic steatohepatitis, arthritis, otitis media and ischemia-reperfusion injuries (Sun et al., 2020; Li et al., 2021; Dou et al., 2022). The key events and pathways of ferroptosis, including iron overload, ROS accumulation, lipid peroxidation and impaired antioxidant systems, are involved in these inflammation-related diseases. Similar to IBD, GPX4 activator treatment inhibits hepatic lipid peroxidation and reduces the diseases severity in the non-alcoholic steatohepatitis mouse model (Qi et al., 2020). Therefore, further research is needed to identify disease-specific ferroptotic mechanisms to develop disease context-dependent therapeutic regimens.

Furthermore, ferroptosis cells interact with inflammatory cells. However, there are still some important questions to be answered. For example, the molecular mechanisms by which immune cells response to ferroptotic cells remains unclear. In addition, it is unknown whether inflammatory cells in inflammatory intestinal diseases themselves undergo ferroptosis and whether they are involved in the progress of inflammation.

Taken together, as a newly discovered form of programmed cell death, ferroptosis provides many new clues to better understand the nature of diseases, and further investigation of ferroptosis may provide novel targets for the treatment of inflammation and related diseases.
